# Clear Cell Carcinomas of the Mullerian System: Does the Pathogenesis Vary Depending on Their Nuclear Grade and Their Association with Endometriosis? An Immunohistochemical Analysis

**DOI:** 10.1155/2012/674748

**Published:** 2012-11-01

**Authors:** Ahmad Alduaij, Katrine Hansen, Tahreem A. Karim, Cunxian Zhang, Michelle M. Lomme, C. James Sung, W. Dwayne Lawrence, M. Ruhul Quddus

**Affiliations:** Department of Pathology, The Warren Alpert Medical School of Brown University and Women & Infants Hospital, 101 Dudley Street, Providence, RI 02905, USA

## Abstract

Clear cell carcinomas (CCC) of the mullerian system are considered high grade tumors, but morphologically, the cells of CCC show both low and high grade features. The aims of the current study were to categorize CCC into low and high nuclear grade types, correlate their association with endometriosis, and then observe possible variations in pathogenesis based on their expression of p53 and Ki-67. We studied 41 pure mullerian CCCs and designated each as either a high (HNG) or low (LNG) nuclear grade tumor. Morphologically, 17 (41%) CCCs were LNG and 24 (59%) were HNG. Nine (38%) HNG and 2 (12%) LNG tumors showed positive immunostaining with p53. Endometriosis was associated with 8 (47%) LNG tumors and 8 (33%) HNG CCCs. Of the 11 cases with p53 alteration, 4 (1 LNG and 3 HNG) were associated with endometriosis. Conclusions: HNG CCCs, irrespective of their association with endometriosis, have alterations of p53. In general, LNG ovarian and endometrial CCCs, irrespective of their association with endometriosis/adenomyosis, are less likely to show p53 alteration. It appears that mullerian CCCs may have variable pathogenesis depending on their nuclear grade and association with endometriosis. A larger study is needed to validate these findings.

## 1. Introduction

While the pathogenesis of mullerian serous and endometrioid carcinomas has been linked to p53 and PTEN mutations, respectively, the pathogenesis of clear cell carcinoma remains largely speculative. Clear cell carcinoma (CCC) was initially termed “mesonephroid tumor” in 1939 [1] and, since 1973, has been strictly defined by the WHO as a tumor characterized by clear cells growing in solid/tubular or glandular patterns and sometimes as hobnail cells [2]. CCCs of the mullerian system are largely found in the endometrium and ovary, but primary peritoneal clear cell carcinoma has also been reported. CCCs also arise in the uterine cervix and vagina of women of all ages including children [3, 4].

It is known that many clear cell carcinomas arise in endometriosis. Recent studies have speculated that ovarian clear cell carcinoma may develop along two pathways, both of which are related to endometriosis [5]. In one pathway, the epithelial atypia arises within an endometriotic cyst and then progresses to carcinoma. In the noncystic pathway, endometriosis induces fibromatosis resulting in the formation of an adenofibroma. In this second pathway, adenofibromas progress to atypical adenofibroma and subsequently to CCC. CCCs of the uterus have been reported to arise from adenomyosis [6]. Studies have shown that cystic CCCs and adenofibromatous CCCs have different clinicopathologic features. Cystic CCC is often diagnosed at an early stage while adenofibromatous CCCs are found at higher stage. Cystic CCCs were more often found in association with atypical endometriosis [7, 8].

Although generally considered an aggressive tumor and uniformly graded as high grade, it is known that some mullerian CCCs have a better prognosis than others [9, 10]. A recent study showed that CCCs with poorly differentiated histology, based largely on architectural features, had a significantly adverse clinical outcome compared with tumors showing no poorly differentiated histology [11]. In addition to differences in architecture, tumor cell nuclei may vary widely between cases. Both cystic and solid CCC may be composed of tumor cells with high nuclear grade, often with bizarre nuclei. Other CCCs are composed of cells with low nuclear grade. It is not known whether LNG and HNG CCCs have different pathogenetic pathways, and different prognoses.

Unlike serous carcinomas of the ovary and endometrium, CCCs are not, in general, considered tumors that show aberrant p53 expression. Studies, however, report a wide range, 9% to as high as 60%, of p53 mutation in CCCs [12–14]. A recent study of endometrial tumors with clear cell histology and p53 expression showed that endometrial CCC with aberrant p53 expression presented as advanced disease [15].

In this study, mullerian CCCs from our institution were classified as either a low nuclear grade tumor or high nuclear grade tumor. The nuclear grade of CCCs was correlated with the presence or absence of endometriosis, as well as p53, and Ki-67 expression. 

## 2. Design

41 pure mullerian CCCs (26 ovarian and 15 endometrial) were retrieved from the archival files of the Department of Pathology, Women and Infants Hospital of Rhode Island, after IRB approval. All the cases included in the study were unequivocal pure CCC's diagnosed based on morphologic features by experienced fellowship trained gynecologic pathologists [16]. The original H and E slides were reviewed by two pathologists to assign nuclear morphologic grading of the tumor cells, that is, low nuclear grade or high nuclear grade. The low nuclear grade tumors had small and mostly uniform nuclei. The high nuclear grade tumors had predominantly larger nuclei (at least twice the size of the LNGs) and often had bizarre nuclear forms. The CCCs were categorized based on the presence of greater than 90% of either the low or high grade nuclei and designated as either LNG or HNG. The presence of endometriosis in ovarian CCCs was noted. For the endometrial CCCs, the presence of adenomyosis or endometriosis elsewhere in the pelvis or abdominal cavity was noted from the history and/or pathology reports. 4 micron sections were cut from 41 cases and p53, and Ki-67 immunohistochemistry was done using an automated Dako immunostainer. The Dako monoclonal MIB 1 was used for ki-67. Positive and negative control slides were run with each batch. All immunostains were blindly evaluated independently and later jointly on a double headed microscope by two pathologists (AA, MRQ) and a consensus was reached regarding the staining pattern. The p53 immunostain was considered positive when the nuclear staining was strong and diffuse. Weak, focal staining was considered negative [17]. The Ki-67 labeling index was determined by counting 500 cells in each specimen. The Ki-67 labeling index was determined with the following formula: LI% = 100 × labeled cells/all cells. A cut-off value for LI was set at 15%. A value less than 15% would indicate a lack of proliferation. 

The nuclear grading of CCCs was correlated with p53 and Ki67 expression and also with endometriosis. Results were tabulated using the sum of the scores obtained from percentage of the cells stained, intensity, and extent of staining. The method has been described in detail elsewhere [18]. 

## 3. Results

**Figure 1:**
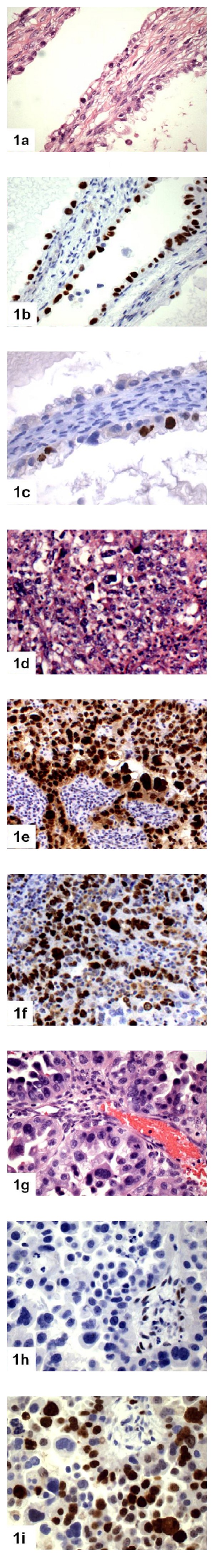
Ovarian low nuclear grade clear cell carcinoma showing p53 and Ki67 positivity (1(a): H&E 10x; 1(b): p53 10x; 1(c): Ki67 10x). Ovarian high nuclear grade CCC showing p53 and Ki67 positivity (1(d): H&E x 20x; 1(e): p53 20x; 1(f): Ki67 20x). Ovarian HNG CCC negative for p53 but positive for Ki67 (1(g): H&E 20x; 1(h): p53 20x; 1(i): Ki67 20x).

**Figure 2:**
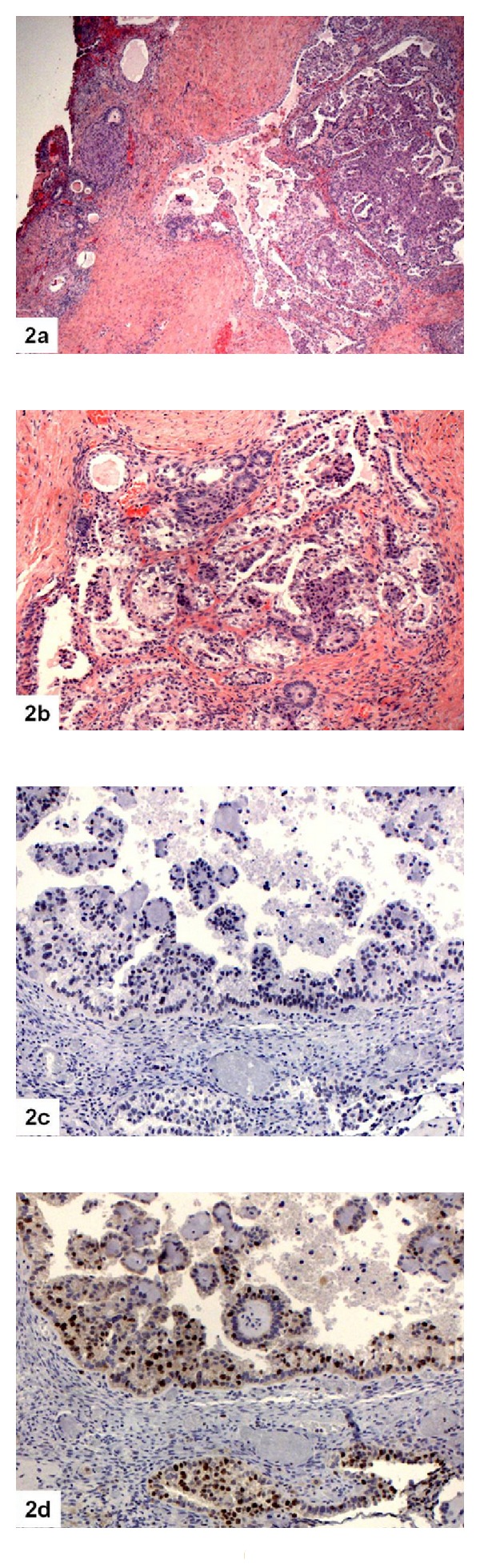
Ovarian low nuclear grade CCC associated with endometriosis showing negative staining for p53 but positive staining for Ki67 (2(a): H&E 4x; 2(b): H&E 10x; 2(c): p53 10x; 2(d): Ki67 10x).

**Figure 3:**
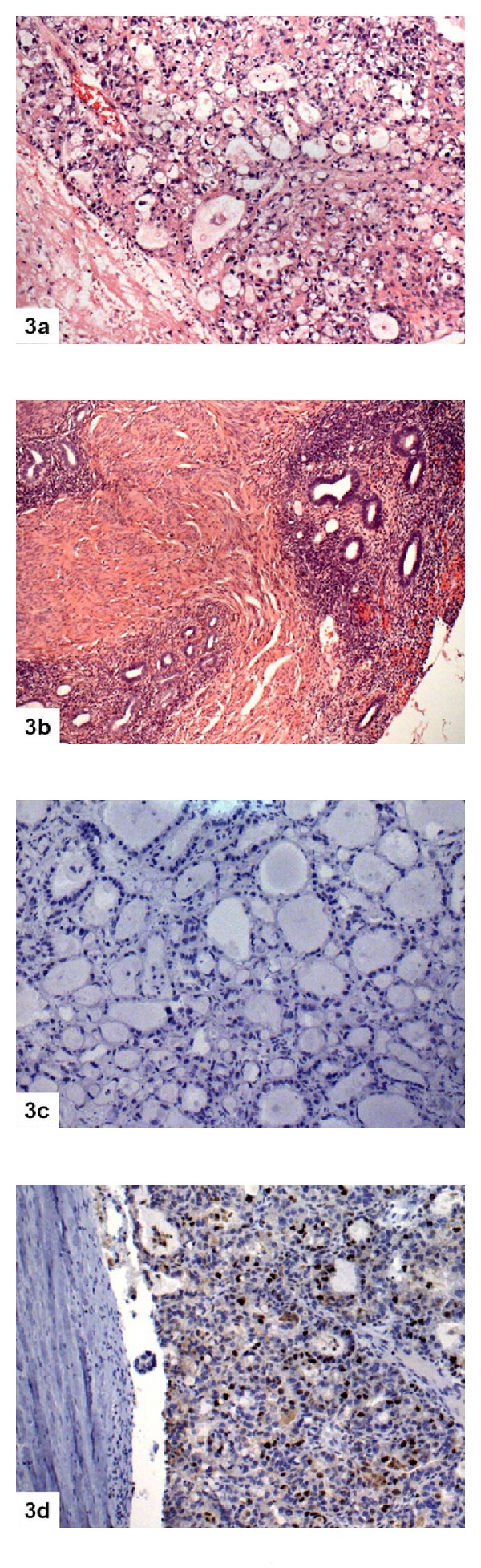
Endometrial low nuclear grade CCC with adenomyosis showing negative staining for p53 and positive staining for Ki67 (3(a): CCC H&E 10x; 3(b): adenomyosis H&E 10x; 3(c): p53 10x; 3(d): Ki67 10x).

41 cases of mullerian CCC were identified; 26 were ovarian and 15 were endometrial in origin. Adequate tissue material was available in all 41 cases; of which 24 cases were assigned as high nuclear grade and 17 were low nuclear grade tumors (Tables 1 and 2). The presence or absence of endometriosis in both groups of tumors were noted in Table 2. p53 expression categorized according to low and high nuclear grades is also shown in Table 2.  Figure 1 shows p53 expression pattern in LNG and HNG CCCs. Proliferative index marker (Ki67) showed brisk positivity (greater than 15% of cells stained in each specimen) in both low and high nuclear grade tumors irrespective of their association with endometriosis (Figure 1). Figures 2 and 3 show p53 and Ki67 expression in ovarian and endometrial LNG CCCs. Tables 3, 4, 5, and 6 include the basic clinical parameters (age and stage) of patients with CCC with and without endometriosis.

## 4. Discussion

The pathogenesis and origin of mullerian clear cell carcinomas remain largely mysterious. Clear cell carcinomas of the mullerian system are seen predominantly in the endometrium and in the ovary. Ovarian CCCs are often associated with endometriosis, and an association with atypical endometriosis is documented [19]. The main focus of this study was to determine if clear cell carcinomas show differential association with endometriosis depending on their overall nuclear grade. Secondarily we looked for possible variations in pathogenesis based on tumor expression of p53 and Ki67. 

We found that 17 (41%) of CCCs were predominantly LNG and 24 cases were HNG (59%). Endometriosis was found associated with 8 of 17 (47%) LNG CCCs and 8 of 24 (33%) HNG tumors. These findings are similar to the reports in the literature where an average of 30–35% of ovarian CCCs is shown to arise in association with endometriosis in the ovary or elsewhere in the pelvis or abdominal cavity [19, 20]. Our data suggests that endometriosis associated CCCs are more likely to be of LNG type.

Positive p 53 staining was noted in 27% of both ovarian and endometrial CCCs. Of the 7 ovarian cases with p53 alteration, 1 was LNG and 6 were HNG. Of these 7 cases, the lone case of LNG and 3 of 6 HNG ovarian CCCs were associated with endometriosis. Among the 4 endometrial CCCS with p53 alteration, 1 was LNG and 3 were HNG tumors. None of these cases were associated with adenomyosis or endometriosis in the pelvis. Adenomyosis or endometriosis in the pelvis does not appear to be a risk factor for endometrial CCCs.

A high index of proliferation was present in all 41 cases of CCC in this study. This result differs from that of Itamochi et al. [21] who reported a group of CCCs with low labeling index associated with lack of chemotherapy response. In the same study it was reported that the Ki67 labeling index in clear cell carcinoma was significantly lower than serous adenocarcinoma, and that the labeling index for chemotherapy responders was significantly higher than that for nonresponders in both tumor types.

## 5. Conclusion

LNG ovarian CCCs arising in endometriosis and uterine CCC s associated with adenomyosis/endometriosis elsewhere in the pelvis are less likely to show p53 alteration. The HNG ovarian CCCs, irrespective of their association with endometriosis, are more frequently associated with underlying p53 alteration. HNG uterine CCCs not associated with adenomyosis are likely to show p53 alteration. All CCCs, irrespective of nuclear grade, showed high Ki67 proliferative index. 

Our study reveals that mullerian CCCs have variable pathogenesis irrespective of their nuclear grade and/or association with endometriosis. Our study, however, is limited by the small number of cases which did not permit us to make statistically significant conclusions. A multicenter study with a larger number of cases is needed in order to validate the findings of this study.

## Figures and Tables

**Table 1:** Distribution of mullerian carcinoma with and without endometriosis.

	Clear cell carcinoma (*N* = 41)				
	Ovarian Origin (*N* = 26)			Endometrial Origin (*N* = 15)	
	With endometriosis (*N* = 13)	Without endometriosis (*N* = 13)		With adenomyosis/endometriosis elsewhere (*N* = 3)	Without adenomyosis/endometriosis elsewhere (*N* = 12)
HNG (*N* = 13)	6	7	HNG (*N* = 11)	2	9
LNG (*N* = 14)	7	6	LNG (*N* = 4)	1	3

**Table 2:** p53 expression clear cell carcinoma of the mullerian system.

			p53 in clear cell carcinoma (*N* = 41)						
	Ovarian origin (*N* = 26)					Endometrial origin (*N* = 15)			
	With endometriosis (*N* = 13)		Without endometriosis (*N* = 13)			With adenomyosis/ endometriosis elsewhere (*N* = 3)		Without adenomyosis/ endometriosis elsewhere (*N* = 12)	
	P53+	P53−	P53+	P53−		P53+	P53−	P53+	P53−
HNG (*N* = 13)	3	3	3	4	HNG (*N* = 11)	0	2	3	6
LNG (*N* = 13)	1	6	0	6	LNG (*N* = 4)	0	1	1	2

**Table 3:** Stage of ovarian CCCs with and without associated endometriosis.

	With endometriosis (*n* = 13)				Without endometriosis (*n* = 13)			
	Stage I	Stage II	Stage III	Stage IV	Stage I	Stage II	Stage III	Stage IV
HNG (*n* = 13)	2	1	3	0	1	2	3	1
LNG (*n* = 13)	5	1	1	0	6	0	0	0

**Table 4:** Stage of endometrial CCCs with and without associated adenomyosis of endometriosis elsewhere.

	With adenomyosis or endometriosis				Without adenomyosis or			
	elsewhere (*n* = 3)				endometriosis elsewhere (*n* = 12)			
	Stage I	Stage II	Stage III	Stage IV	Stage I	Stage II	Stage III	Stage IV
HNG (*n* = 11)	2	0	0	0	4	1	4	
LNG (*n* = 4)	1	0	0	0	2	0	1	0

**Table 5:** Age range, mean, and median of ovarian CCCs with and without endometriosis.

	Ovarian CCCs with endometriosis			Ovarian CCCs without endometriosis		
	Age range (yrs)	Mean (yrs)	Median (yrs)	Age range (yrs)	Mean (yrs)	Median (yrs)
HNG (*n* = 13)	52–81	64.6	63.5	42–76	59.8	59
LNG (*n* = 13)	39–74	57.8	62	38–77	61.5	62.5

**Table 6:** Age range, mean, and median of uterine CCCs with and without adenomyosis or endometriosis elsewhere.

	Endometrial CCCs with			Endometrial CCCs without		
	endometriosis (*n* = 3)			adenomyosis or endometriosis elsewhere (*n* = 12)		
	Age range (yrs)	Mean (yrs)	Median (yrs)	Age range (yrs)	Mean (yrs)	Median (yrs)
HNG (*n* = 11)	53–58	55.5	55.5	70–97	79.4	77
LNG (*n* = 4)	72	72	72	65–86	73.6	70

## References

[1] Schiller W (1939). Mesonephroma ovarii. *American Journal of Cancer*.

[2] Serov SF, Scully RE, Sobin LH (1973). International histologic classification of tumors. *Histologic Classification of Tumors*.

[3] Van Der Aa MA, Helmerhorst TJM, Siesling S, Riemersma S, Coebergh JW (2010). Vaginal and (uncommon) cervical cancers in the Netherlands, 1989–2003. *International Journal of Gynecological Cancer*.

[4] Lester FC, Farmer DL, Rabban JT, Chen LM (2010). Radical trachelectomy for clear cell carcinoma of the cervix in a 6-year old: a case report, review, and description of the surgical technique. *Journal of Pediatric Surgery*.

[5] Zhao C, Wu LS, Barner R (2011). Pathogenesis of ovarian clear cell adenofibroma, atypical proliferative (borderline) tumor, and carcinoma: clinicopathologic features of tumors with endometriosis or adenofibromatous components support two related pathways of tumor development. *Journal of Cancer*.

[6] Kashiyama M, Suzuki A, Ozawa M (2002). Adenocarcinomas arising from uterine adenomyosis. *International Journal of Gynecological Pathology*.

[7] Terada T (2011). Clear cell adenocarcinoma of the ovary arising in atypical endometriosis: a report of eight cases. *Archives of Gynecology and Obstetrics*.

[8] Veras E, Mao TL, Ayhan A (2009). Cystic and adenofibromatous clear cell carcinomas of the ovary: distinctive tumors that differ in their pathogenesis and behavior: a clinicopathologic analysis of 122 cases. *American Journal of Surgical Pathology*.

[9] Carcangiu ML, Chambers JT (1995). Early pathologic stage clear cell carcinoma and uterine papillary serous carcinoma of the endometrium: comparison of clinicopathologic features and survival. *International Journal of Gynecological Pathology*.

[10] Malpica A, Tornos C, Burke TW, Silva EG (1995). Low-stage clear-cell carcinoma of the endometrium. *American Journal of Surgical Pathology*.

[11] Yamamoto S, Tsuda H, Shimazaki H (2011). Clear cell adenocarcinoma with a component of poorly differentiated histology: a poor prognostic subgroup of ovarian clear cell adenocarcinoma. *International Journal of Gynecological Pathology*.

[12] Reles A, Schmider A, Schönborn I (1996). Immunostaining of p53 protein in ovarian carcinoma: correlation with histopathological data and clinical outcome. *Journal of Cancer Research and Clinical Oncology*.

[13] Lax SF, Pizer ES, Ronnett BM, Kurman RJ (1998). Clear cell carcinoma of the endometrium is characterized by a distinctive profile of p53, Ki-67, estrogen, and progesterone receptor expression. *Human Pathology*.

[14] An HJ, Logani S, Isacson C, Ellenson LH (2004). Molecular characterization of uterine clear cell carcinoma. *Modern Pathology*.

[15] Delair D, Soslow RA (2012). Endometrial clear cell carcinomas with and without aberrant p53 expression: a study of 16 cases. *Modern Pathology*.

[16] Fadare O, Parkash V, Dupont WD (2012). The diagnosis of endometrial carcinoma with clear cells by gynecologic pathologists: an assessment of interobserver variability and associated morphologic features. *The American Journal of Surgical Pathology*.

[17] Yemelyanova A, Vang R, Kshirsagar M (2011). Immunohistochemical staining patterns of p53 can serve as a surrogate marker for TP53 mutations in ovarian carcinoma: an immunohistochemical and nucleotide sequencing analysis. *Modern Pathology*.

[18] Quddus MR, Sung CJ, Zheng W, Lauchlan SC (1999). p53 immunoreactivity in endometrial metaplasia with dysfunctional uterine bleeding. *Histopathology*.

[19] Fukunaga M, Nomura K, Ishikawa E, Ushigome S (1997). Ovarian atypical endometriosis: its close association with malignant epithelial tumours. *Histopathology*.

[20] Toki T, Fujii S, Silverberg SG (1996). A clinicopathologic study on the association of endometriosis and carcinoma of the ovary using a scoring system. *International Journal of Gynecological Cancer*.

[21] Itamochi H, Kigawa J, Sugiyama T, Kikuchi Y, Suzuki M, Terakawa N (2002). Low proliferation activity may be associated with chemoresistance in clear cell carcinoma of the ovary. *Obstetrics and Gynecology*.

